# Nocturnal Oxygen Saturation Parameters as Independent Risk Factors for Type 2 Diabetes Mellitus among Obstructive Sleep Apnea Patients

**DOI:** 10.3390/jcm10173770

**Published:** 2021-08-24

**Authors:** Agata Gabryelska, Jędrzej Chrzanowski, Marcin Sochal, Piotr Kaczmarski, Szymon Turkiewicz, Marta Ditmer, Filip Franciszek Karuga, Leszek Czupryniak, Piotr Białasiewicz

**Affiliations:** 1 Department of Sleep Medicine and Metabolic Disorders, Medical University of Lodz, 92-215 Lodz, Poland; sochalmar@gmail.com (M.S.); piotr.kaczmarski@stud.umed.lodz.pl (P.K.); szymon.turkiewicz@stud.umed.lodz.pl (S.T.); marta.ditmer@stud.umed.lodz.pl (M.D.); filip.karuga@stud.umed.lodz.pl (F.F.K.); piotr.bialasiewicz@umed.lodz.pl (P.B.); 2 Department of Biostatistics and Translational Medicine, Medical University of Lodz, 92-215 Lodz, Poland; jedrzej.chrzanowski@umed.lodz.pl; 3 Department of Diabetology and Internal Medicine, Warsaw Medical University, 02-097 Warsaw, Poland; leszek.czupryniak@wum.edu.pl

**Keywords:** obstructive sleep apnea (OSA), type 2 diabetes mellitus (DM2), polysomnography (PSG), O_2_ saturation, hypoxia, risk factor

## Abstract

Obstructive sleep apnea (OSA) is a recognized independent risk factor for metabolic disorders, type 2 diabetes mellites (DM2) in particular. Therefore, the study aimed to assess the influence of nocturnal oxygen saturation parameters on the onset of DM2 among OSA patients. The study consisted of 549 participants, who underwent polysomnography examination. Based on apnea hypopnea index (AHI), 465 patients were diagnosed with OSA. One hundred and seven individuals had comorbid DM2. Cox regression models were used to assess the effect of oxygen saturation parameters on the onset of DM2. Classification and regression trees (CART) analysis was used to assess the onset of the DM2 in the study group in context of oxygen saturation variables. One-way Cox regression showed higher risk of earlier DM2 for increased values of BMI, AHI, decreased basal O_2_ and O_2_ nadir value, while lowered mean O_2_ desaturation has not shown statistical significance. In the CART analysis, the following cut-off points 92.2%, 81.7%, 87.1% were determined for basal O_2_, O_2_ nadir and mean O_2_ desaturation, respectively, with the first two parameters being statistically significant. Therefore, basal O_2_ is independent from AHI, BMI and age is a risk factor of DM2 among OSA patients.

## 1. Introduction

Type 2 diabetes mellitus (DM2) is one of the most prevalent civilizational diseases and is associated with great morbidity and mortality [1]. It is important to identify potentially modifiable risk factors for DM2. Obstructive sleep apnea (OSA) is a common sleep respiratory disease characterized by repetitive collapse of upper airways resulting in sleep fragmentation and nocturnal recurrent intermittent hypoxia (IH), which manifests as desaturations in polysomnography (PSG). Prevalence estimation shows that around one billion adults worldwide could have OSA (apnea—hypopnea index [AHI] ≥ 5/h) [2]. Considering the scale of the problem it is important to properly recognize and treat many chronic medical conditions associated with OSA such as hypertension, coronary artery disease and metabolic disorders [3,4]. Numerous studies have provided evidence that OSA may be independent from other classic risk factors as a determinant for incident DM2 [5,6,7]. Intermittent hypoxia in OSA-related respiratory disorders may cause many metabolic disturbances such as insulin resistance and an onset of DM2 [8]. The exact mechanism of this connection is not yet known, but possible pathophysiological pathways may be involved, such as hypoxia-inducible factor 1α (HIF-1α) [9], key regulator of oxygen metabolism, as the factor is upregulated in OSA patients [10,11,12,13]. Few authors have connected the fact of nocturnal hypoxemia and its physiological consequences (e.g., oxygen desaturation) and possibility of increased risk of DM2 [14]. There is an important need to evaluate which parameters are essential to assess the risk of onset of metabolic disturbances due to nocturnal hypoxemia. Monitoring oxygen saturation parameters (SpO_2_ basal, mean O_2_ desaturation, SpO_2_ nadir) is a routine procedure during PSG which is a gold-standard procedure for diagnosis of OSA. There is not enough evidence of how nocturnal saturation parameters may be correlated with the DM2. There is some literature reporting that nocturnal hypoxemia has an impact on impaired glucose metabolism and higher HbA1c levels in individuals with and without OSA [15,16]. Therefore, the purpose of this study was to assess the utility of saturation parameters as a predictive factor for incident DM type 2 among OSA patients.

## 2. Materials and Methods

The retrospective study included data from patients being evaluated at the Department of Sleep Medicine and Metabolic Disorders of Medical University of Lodz with presumptive diagnosis of OSA (between January 2017 and February 2020). All patients included in the study were assessed and investigated by authors and underwent diagnostic PSG; while scoring PSG studies, the authors were blinded for the clinical data. All patients gave their informed consent for the sleep study. Demographic and clinical information was collected from patients’ histories, including taken medications, other diseases, in particular, DM2. The following inclusion criteria were applied in the study: age 18–70 and body mass index (BMI) 20–45 kg/m^2^ patients were excluded from the study if their total sleep time was shorter than three hours, if sleep time either in lateral or supine position was shorter than half an hour or if total REM sleep was shorter than half an hour. Moreover, patients diagnosed with any chronic respiratory conditions (e.g., bronchial asthma, or chronic obstructive pulmonary disease) and any sleep disorders other than OSA (e.g., insomnia, delayed phase syndrome) were excluded from the study. Furthermore, exclusion criteria included chronic inflammatory diseases (e.g., connective tissue diseases or inflammatory bowel diseases), diagnosis of cancer (active or in medical history), psychiatric disorders and shift work system, jet lag due to a flight within 2 weeks of the study or taking medications affecting sleep (e.g., benzodiazepines and melatonin).

### 2.1. Polysomnography

Patients were admitted to the sleep lab at 21:00 h (±0.5 h) and underwent physical examination (measurement of body mass, height, heart rate and blood pressure). A standard nocturnal polysomnography was performed by recording the following channels: electroencephalography (C4\A1, C3\A2), chin muscles and anterior tibialis electromyography, electrooculography, measurements of oro-nasal air flow (a thermistor gauge), snoring, body position, respiratory movements of chest and abdomen (piezoelectric gauges), unipolar electrocardiogram and haemoglobin oxygen saturation (SpO_2_) (Alice 6, Phillips-Respironics). Sleep stages were scored according to the criteria based on 30 s epoch standard [17]. Apnea was attained with the reduction of air flow to less than 10% of the baseline for at least 10 s. Hypopnea was defined as at least 30% reduction of air flow for at least 10 s, accompanied by over 3% decrease in SpO_2_ or an arousal. Encephalography arousals were scored according to the American Academy of Sleep Medicine guidelines [17]. The study was conducted in accordance with the amended Declaration of Helsinki.

### 2.2. Statistical Analysis

Statistical analyses were performed using Statistica 13.3 (Statsoft, TIBCO). Due to the lack of normality of continuous variables, they were compared using non-parametric tests. To analyze differences between continuous variables between two groups, the Mann–Whitney U test for independent groups was used. In case of more than two groups being compared, generalized linear models and Kruskall–Wallis tests were performed. For nominal variables, we applied Pearson’s chi-squared test.

To compare the influence of factors on hazard of DM2 development, Cox’s regression was applied. For multivariate regression, forward stepwise feature selection was applied to determine features included in a multivariate model—BMI, AHI and basal O_2_ were selected. The Kaplan–Meier curves for the age of DM2 development using log-rank test were also compared. The groups for Kaplan-Meier curves analysis were determined using classification and regression trees for basal O_2_, O_2_ mean desaturation and O_2_ nadir level. Alfa level for statistical comparisons at level 0.05 was assumed to be significant. Bonferroni–Holm correction to maintain false discovery rate below 25% was applied.

## 3. Results

**Figure 1:**
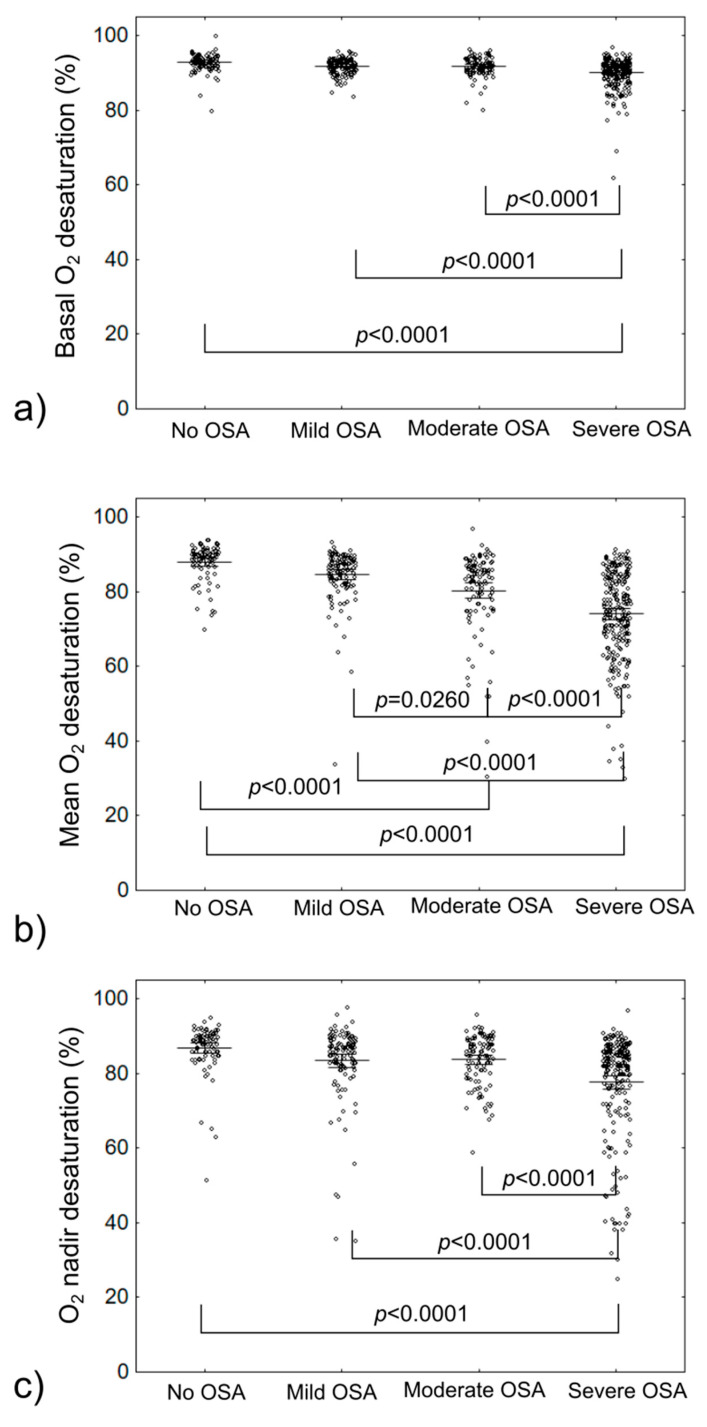
Comparison of O_2_ saturation parameters in different OSA severity groups; (**a**)—based on basal O_2_ saturation; (**b**)—based on Mean O_2_ desaturation; (**c**)—based on O_2_ nadir desaturation; OSA—obstructive sleep apnea.

Altogether, 549 patients were included in the analysis, with median age 62 (IQR: 57–68). General group characteristics are provided in Table 1. Significant correlation between BMI, AHI values and basal O_2_, mean desaturation O_2_ and O_2_ nadir level evaluated during PSG were observed. Further results of comparison of basal O_2_, mean desaturation O_2_ and O_2_ nadir level (unadjusted and adjusted for BMI and AHI) between patients with and without OSA, and for different OSA severity are provided in Table 2 and on Figure 1. Equations for adjustments are provided in Supplementary Table S1.

**Figure 2:**
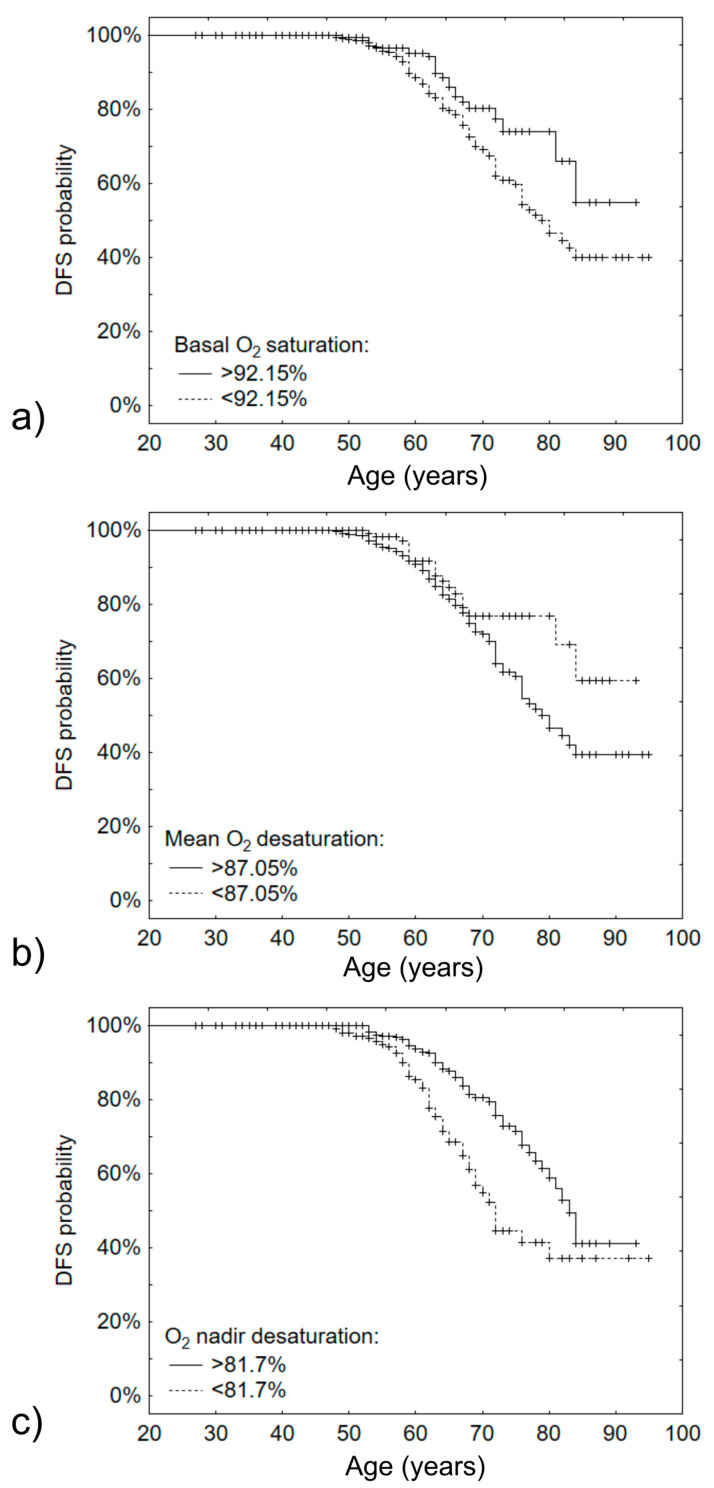
Kaplan–Meier curves for development age of DM2 based on O_2_ saturation parameters: (**a**)—based on basal O_2_ saturation, cut-off point 92.15%; (**b**)—based on mean O_2_ desaturation, cut-off point 87.05%; (**c**)—based on O_2_ nadir desaturation, cut-off point 82.7%.

Next, the effects of BMI, AHI, basal O_2_, mean desaturation O_2_ and O_2_ nadir level on the age of DM2 development were determined. Univariate and multivariate analyses have shown that BMI, AHI, basal O_2_ and O_2_ nadir level are significantly altered in patients developing DM2 at a younger age (Table 3). Using classification and regression trees, we determined that basal O_2_ < 92.15% and O_2_ nadir level < 81.7% were effective for separation of patients at higher risk of DM2 at a younger age (log-rank p-value 0.0161 and 0.0001 respectively). Kaplan–Maier curves are provided in Figure 2.

## 4. Discussion

This retrospective study showed that desaturation parameters assessed by polysomnography examination are associated with increased risk of DM2. Higher SpO_2_ nadir and basal SpO_2_ are correlated with the later onset of DM2 in OSA patients while basal O_2_ is independent from AHI, BMI and age predictor of DM2 among OSA patients. The current study provides convincing evidence for correlation between nocturnal hypoxemia in the course of OSA and early onset of DM2.

The results of our research are consistent with other existing literature on that subject. In this field of research there has been a lot of OSA-related factors taken under consideration as possible predictors for DM2. A prospective analysis of Atherosclerosis Risk in Communities Study and the Sleep Heart Health Study proves that the severity of OSA assessed by AHI is an independent risk factor of incidence of DM2 [6]. Another clinical cohort study provided evidence that not only severity of OSA but also REM-AHI, duration of O_2_ saturation less than 90%, shorter total sleep time, higher mean heart rate, greater neck circumference, and the presence of daytime sleepiness were significant predictors of DM2 [14].

An interesting hypothesis of connection between OSA and DM2 is that parameters of oxygenation may be responsible for different manifestations and comorbidities of OSA independently from AHI [18]. Desaturation parameters such as SpO_2_ nadir assessed by pulse oximetry has also been associated with the risk of DM2 in Japanese prospective study [19]. However, the lack of polysomnography is the main limitation of this study.

Nocturnal intermittent hypoxia is characteristic for OSA patients. There are a few studies showing that hypoxemia and its reflection in desaturation parameters in PSG may be corelated with increased level of HbA1c [16,20], and other metabolic disturbances such as dyslipidemia [21].

Our findings match the proposed pathophysiological pathways linking nocturnal hypoxia and DM2. There are several mechanisms that can possibly lead to metabolic disorders including intermittent hypoxia, sleep fragmentation, elevated sympathetic tone, and oxidative stress [22]. In addition to the previously mentioned hypothesis, there is a new interesting pathway including HIF 1α, a factor that plays a significant role in the glucose metabolism in hypoxemic condition and may be responsible for insulin resistance and the development of diabetes [9]. Moreover, upregulation of HIF-1α alone may be considered as an independent risk factor for OSA [11].

We are aware of several limitations of our study that should be acknowledged in interpretation of the results, such as lack of fasting glucose of glycated hemoglobin evaluation as some individuals might have suffered from not-yet-diagnosed DM2; however, this should not greatly influence the obtained results, especially considering that the large group of patients is representative of the general population. Furthermore, our analysis consisted of more than one oximetric parameter and the method of recording these parameters (full-night PSG) which are the strengths of this research.

The results of our research and many literature reports may lead to a conclusion that monitoring nocturnal oximetric parameters may be useful for recognizing patients at risk of developing metabolic disorders. This thesis is reflected in recommendations of the International Diabetes Federation Taskforce on Epidemiology and Prevention for the health professionals working with both DM2 and OSA patients [23]. In clinical practice, considering patients with one condition for the other should be adopted. Despite the number of convincing scientific reports in that field, further research is needed to examine this correlation in a prospective study. Especially including assessment of CPAP treatment and compering other chronic pulmonary disorders that might present with hypoxia such as chronic obstructive pulmonary diseases and cystic fibrosis [24].

## Figures and Tables

**Table 1:** Group characteristics.

Parameter	All Participants	No OSA (*n* = 155; AHI < 5)	OSA (*n* = 394; AHI ≥ 5)	*p*-Value
Age (years)	62.00 (57.00–68.00)	61 (53.5–69)	62 (57–68)	0.1525
Sex (male)	56.60%	47.62%	58.28%	0.0696
BMI (kg/m^2^)	31.13 (27.46–35.66)	28.69 (25.61–31.65)	31.56 (27.92–36.20)	<0.0001
AHI (events/h)	24.30 (9.00–49.50)	2.1 (1–3.75)	30.38 (14.3–52.89)	<0.0001
Basal O_2_ (%)	91.90 (90.00–93.20)	93.2 (91.75–94.05)	91.6 (90–93)	<0.0001
Mean desaturation O_2_ (%)	83.45 (74.10–88.00)	89.8 (86.9–91)	82 (72.9–87)	<0.0001
O_2_ nadir (%)	85.00 (79.90–88.10)	88.9 (85–90.5)	84 (78.1–87.9)	<0.0001
DM2	19.50%	10.71%	21.08%	0.0274

AHI—apnea–hypopnea index, BMI—body mass index, DM2—diabetes mellitus type 2, OSA—obstructive sleep apnea.

**Table 2:** Comparison of O_2_ saturation parameters in different OSA severity groups.

	Groups						
Saturation Parameter	No OSA (AHI < 5)	Mild OSA (15 > AHI ≥ 5)	Moderate OSA (30 > AHI ≥ 15)	Severe OSA (AHI ≥ 30)	Any OSA (AHI ≥ 5)	*p*-Value	*p*-Value Adjusted
Basal O_2_ (%)	93.20 (91.75–94.05)	92.00 (90.90–93.40)	92.00 (90.90–93.40)	91.00 (88.80–92.30)	91.6 (90.00–93.00)	<0.0001 <0.0001 *	<0.0001 <0.0001 *
Mean desaturation O_2_ (%)	89.80 (86.90–91.00)	86.50 (82.40–88.90)	83.95 (76.90–87.00)	75.25 (67.00–83.90)	82.00 (72.90–87.00)	<0.0001 <0.0001 *	<0.0001 <0.0001 *
O_2_ nadir (%)	88.90 (85.00–90.50)	86.00 (82.10–88.90)	85.10 (79.90–89.00)	82.10 (75.00–86.20)	84.00 (78.10–87.90)	<0.0001 <0.0001	<0.0001 <0.0001

AHI—apnea–hypopnea index, OSA—obstructive sleep apnea. First *p*-value corresponds to KW *p*-value for No OSA vs. Mild/Moderate/Severe OSA. Second p-value indicated by * corresponds to UMW *p*-value for No vs. Any OSA. Adjusted *p*-value corresponds to difference after adjustment for BMI and AHI values.

**Table 3:** Univariate and multivariate Cox regression analysis for development of DM2 at younger age.

	Univariate Regression			Multivariate Regression		
	HR	95%CI	*p*	HR	95%CI	*p*
BMI	1.1170	1.0826–1.1526	<0.0001	1.1219	1.0824–1.1629	<0.0001
AHI	1.0107	1.0036–1.0178	0.0032	1.0054	0.9980–1.0129	0.1542
Basal O_2_	0.9326	0.8875–0.9799	0.0057	1.0262	0.9494–1.1091	0.5146
Mean deasturatiom O_2_	0.9864	0.9729–1.0002	0.0531	-	-	-
O_2_ nadir	0.9844	0.9727–0.9961	0.0092	-	-	-

AHI—apnea–hypopnea index, BMI—body mass index, CI—confidence interval, HR—hazard ratio.

## Supplementary Materials

The following are available online at https://www.mdpi.com/article/10.3390/jcm10173770/s1, Supplementary Table S1: Equations used for adjustment of basal O_2_ saturation, mean O_2_ desaturation and O_2_ nadir by BMI and AHI based on general linear regression.
