# Supplementary material for: Nocturnal Oxygen Saturation Parameters as Independent Risk Factors for Type 2 Diabetes Mellitus among Obstructive Sleep Apnea Patients
Source: J Clin Med. 2021 Aug 24;10(17):3770. doi: 10.3390/jcm10173770 (PMC8432118; doi:10.3390/jcm10173770)
Supplement: Supplementary file 1 [file jcm-10-03770-s001.zip › jcm-1327940-Supplementary Material.pdf]

Supplementary Table 1. Equations used for adjustment of basal O_2 saturation, mean O_2 desaturation and O_2 nadir by BMI and AHI based on general linear regression.

Parameter	BMI		AHI	
	Beta	p-value	Beta	p-value
Basal O2 [%]	-0.3314	<0.0000	-0.2722	<0.0000
Mean desaturation O2 [%]	-0.1187	0.0033	-0.4805	<0.0000
O2 nadir [%]	-0.2616	<0.0000	-0.2156	<0.0000

AHI – apnea-hypopnea index, BMI – body-mass index
